# Activated Protein C and the Retina: From Physiology to Therapeutic Potential

**DOI:** 10.3390/ijms27052282

**Published:** 2026-02-28

**Authors:** Alon Zahavi, Sarina Levy-Mendelovich, John H. Griffin, Tami Livnat

**Affiliations:** 1 Ophthalmology Department and Laboratory of Eye Research, Felsenstein Medical Research Center, Rabin Medical Center, Petah-Tikva 4941492, Israel; alonzahavi@gmail.com; 2 Gray Faculty of Medical & Health Sciences, Tel Aviv University, Tel Aviv 6997801, Israel; 3 The Amalia Biron Research Institute of Thrombosis & Hemostasis, Gray Faculty of Medical & Health Sciences, Tel Aviv University, Tel Aviv 6997801, Israel; sarina.levy@sheba.health.gov.il; 4 National Hemophilia Center, Thrombosis & Hemostasis Institute, Sheba Medical Center, Ramat Gan 52621, Israel; 5 Department of Translational Medicine, The Scripps Research Institute, La Jolla, CA 92037, USA; griffin@scripps.edu

**Keywords:** protein C, activated protein C (APC), blood–retina barrier (BRB), endothelial protein C receptor (EPCR), retina

## Abstract

Protein C (PC) and its activated form, activated protein C (APC), are well-established regulators of coagulation and cytoprotection. While their systemic functions are extensively characterized, their physiological roles in the retina have only recently begun to be explored. This gap persists despite the observation that congenital PC deficiency is consistently associated with severe ocular complications. Emerging evidence, including the development of a murine model of severe protein C deficiency (SPCD), indicates that APC contributes to retinal integrity and vascular homeostasis under physiological conditions. Beyond its physiological function, APC has shown therapeutic activity in several models of retinal disease. Recent findings from our group further demonstrated that intravenously administered APC and its cytoprotective analog, 3K3A-APC, can cross the blood–retina barrier via the endothelial protein C receptor (EPCR), despite their relatively large molecular weight (~62 kDa), and induce cytoprotective activities in the retina. These findings highlight the translational potential of 3K3A-APC and support its further development as a systemically delivered therapeutic approach for retinal pathologies. This review integrates current knowledge of the molecular biology of the PC/APC pathways with its emerging physiological functions in the retina, and the accumulating preclinical and early clinical evidence that supports its therapeutic relevance.

## 1. Introduction

The retina is a highly specialized extension of the central nervous system that depends on a tightly regulated microenvironment for proper function. This homeostasis is maintained by the blood–retina barrier (BRB), which consists of the inner BRB formed by non-fenestrated endothelial cells and the outer BRB formed by the retinal pigment epithelium. Together, these barriers tightly control the passage of molecules between the circulation and retinal tissue, enabling normal visual processing while protecting the eye from toxins and systemic fluctuations [1,2]. Breakdown of this barrier is a key event in several major blinding diseases, including diabetic retinopathy, age-related macular degeneration (AMD), central retinal vein occlusion (CRVO), and uveitis [2,3].

The physiological role of Protein C (PC) and its active form, activated protein C (APC), in the retina has been poorly explored. This knowledge gap is striking, as both complete and partial congenital PC deficiency are consistently associated with diverse ocular manifestations that cannot be explained solely by thrombosis [4,5,6,7]. Beyond its physiological role, APC has also emerged as a molecule with broad therapeutic potential. In multiple preclinical studies, administration of exogenous APC, including the signaling-selective variant 3K3A-APC, has been shown to confer potent neuroprotective, anti-inflammatory, and tissue-regenerative effects across diverse pathological models [8,9]. Cytoprotective effects have likewise been observed in ocular models, suggesting that the multi-target, multi-action activity of APC extends to the retina and may hold promise for the treatment of retinal vascular and neurodegenerative diseases [10,11]. Altogether, the growing body of evidence suggests that APC contributes to the coordination of vascular and neuroprotective signaling pathways in the retina.

This review summarizes the molecular background of the PC/APC pathway, examines its roles in retinal health and disease, reviews evidence from preclinical and clinical studies of APC-based therapies, and highlights emerging insights into EPCR-mediated transport as a mechanism enabling systemic delivery of APC to the retina.

## 2. Protein C and Activated Protein C Structure and Function

**Figure 1:**
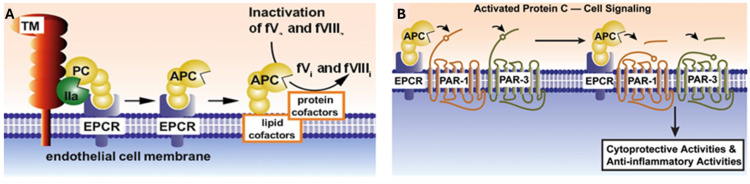
Protein C (PC) activation and expression of multiple biologic activities of activated protein C (APC). (**A**) Proteolytic activation of PC that is bound to endothelial protein C receptor (EPCR) is due to cleavage by thrombin (IIa) that is bound to thrombomodulin (TM) on the endothelial cell membrane. APC’s anticoagulant activity involves its proteolytic inactivation of coagulation factors Va (fVa) and VIIIa (fVIIIa), which is enhanced by lipid and protein cofactors. (**B**) APC’s cell signaling actions often involve its proteolytic activation of two G protein coupled receptors, protease activated receptor (PAR) 1 and PAR3. Cleavage of the extracellular N-terminal PAR tails liberates new N-termini, which can then interact with the receptors to agonize intracellular signaling via beta-arrestin2 that can produce diverse types of cytoprotective activities, which include anti-inflammatory and anti-apoptotic activities and endothelial barrier stabilization [8,9].

Protein C (PC) is a vitamin K-dependent plasma glycoprotein synthesized primarily in the liver and circulates as a two-chain zymogen with a molecular weight of approximately 62 kDa. The mature protein consists of a light chain (≈21 kDa) and a heavy chain (≈41 kDa) linked by a single disulfide bond. The light chain contains the γ-carboxyglutamic acid (Gla) domain, responsible for calcium-dependent binding to negatively charged phospholipid membranes, followed by two epidermal growth factor (EGF)–like domains that mediate high-affinity interaction with the EPCR. The heavy chain comprises the activation peptide and the serine protease domain, which harbors the catalytic triad (His-Asp-Ser) essential for its enzymatic protease activity [12,13]. Activation of PC occurs on the vascular endothelial surface and requires a tightly regulated multiprotein complex (Figure 1A). Thrombin, bound to its cofactor thrombomodulin (TM), cleaves PC at the Arg169–Leu170 bond within the activation peptide, generating APC [13]. This reaction is markedly accelerated, up to 20-fold, when PC is bound to the EPCR, which positions the PC zymogen for efficient cleavage by the thrombin–TM complex [14].

The active serine protease, APC, is best known for its anticoagulant function through proteolytic inactivation of factors Va and VIIIa that is enhanced by lipid and protein cofactors, e.g., negatively charged phospholipid surfaces and protein S (Figure 1A) [8,15]. This anticoagulant activity requires APC dissociation from EPCR, as receptor binding inhibits phospholipid interaction [16,17].

Beyond hemostasis, APC exerts pleiotropic cytoprotective effects, including anti-inflammatory, endothelial barrier-stabilizing, anti-apoptotic, and neuroprotective actions [8,9]. These diverse cytoprotective activities of APC are independent of its anticoagulant function and are primarily mediated by activation of two G protein-coupled receptors (GPCRs), protease-activated receptor (PAR) 1 and PAR3 (Figure 1B). For APC’s biased PAR1-dependent signaling [8], the multifunctional intracellular scaffold protein β-arrestin 2, which associates with PAR1’s intracellular C-terminus, is needed [18,19]. Notably, β-arrestin 2 can mediate multiple diverse signaling responses by employing different effectors to achieve APC’s cytoprotective effects, and in vitro studies identified a variety of signaling nodes and pathways that can be altered by APC-initiated signaling [20,21]. A recent in vivo study showed that β-arrestin 2 is required for APC’s neuroprotection in murine ischemic stroke [22].

APC’s anticoagulant functioning depends on the inactivation of factor Va. Substrate recognition of factor Va by protease-domain exosites, including the Lys191–Lys193 basic loop, is essential, such that when these three lysine (K) residues were replaced with alanine (A) residues in the recombinant 3K3A-APC variant, the anticoagulant activity was reduced by 95% [23]. However, mutation of these three lysine residues had no significant effect on APC’s cytoprotective action, so the 3K3A-APC variant is highly cytoprotective-selective and its use for therapy carries no increased bleeding risk as shown in clinical trials [24,25].

## 3. Protein C Deficiency

The initial insights into the physiologic functioning of PC/APC in humans came from the discovery of mild PC deficiency associated with adult venous thrombosis [26], and of homozygous PC deficiency associated with neonatal purpura fulminans, a severe condition in which infants present within the first days of life with massive venous thrombosis and inflammatory skin necrosis [27].

The molecular mechanisms underlying PC deficiency vary depending on the type of PROC gene mutation. Extensive genetic studies have demonstrated that PROC mutations may lead to either partial or severe loss of APC function [28,29]. Type I PC deficiency is characterized by reduced PC levels due to impaired synthesis or secretion, whereas Type II deficiency is defined by normal PC levels with reduced functional activity. Severe congenital PC deficiency, typically caused by homozygous or compound heterozygous PROC mutations, is associated with PC activity levels below 1% of normal [30,31]. Importantly, in families of infants presenting with lethal purpura fulminans, heterozygous PC-deficient relatives may remain asymptomatic and never develop venous thrombosis [32].

Severe protein C deficiency (SPCD) is most often diagnosed shortly after birth. In some cases, SPCD can be detected already in utero, presenting with cerebral infarctions that may lead to congenital blindness [4]. Delayed clinical presentation has also been reported in adolescents and adults with moderately severe PC deficiency [33].

Long-term management of SPCD requires indefinite anticoagulation, together with replacement therapy using fresh frozen plasma (FFP) or PC concentrates, which are not universally available [30]. Because PC is synthesized by hepatocytes, liver transplantation may provide a definitive cure; however, this approach is feasible only in selected patients and necessitates lifelong immunosuppression following successful transplantation, which entails its complications [34].

## 4. Ocular Manifestations of Protein C Deficiency: Clinical Features and Pathophysiology

Ocular manifestations of PC deficiency have traditionally been primarily related to its prothrombotic effects, which can lead to vascular occlusions and hemorrhagic events in the eye. The most well-documented manifestations include central retinal vein occlusion, transient monocular vision loss due to arterial or venous thrombotic events, and, in severe congenital cases, vitreous hemorrhage [35,36,37]. In heterozygous PC deficiency, ocular complications are rare and typically present later in life, often as isolated thrombotic events such as retinal vein occlusion or transient vision loss. In contrast, homozygous deficiency can present in the neonatal period with severe thromboembolic complications. It is important to note that not all individuals with PC deficiency will develop ocular manifestations, and some familial studies have shown normal retinal vasculature despite the deficiency [38]. However, recurrent or unexplained vascular events in the eye, especially in young patients, should prompt evaluation for underlying hypercoagulable states such as PC deficiency. Most ocular manifestations of PC deficiency arise from microvascular thrombosis and the resulting ischemia–reperfusion injury. As ocular tissues are highly vascularized, they are particularly susceptible to thrombotic occlusion and its secondary consequences [7,39]. Retinal vessels are especially affected: thrombosis of retinal arterioles and venules can precipitate ischemia, triggering compensatory neovascularization and, ultimately, tractional retinal complications [40,41]. The ensuing vitreous hemorrhage from fragile neo-vessels provides a scaffold for fibrovascular proliferation, further exacerbating tractional forces within the vitreous cavity [39]. In some cases, these processes lead to secondary glaucoma, caused by elevated intraocular pressure resulting from neovascularization, vitreous hemorrhage, or inflammatory obstruction of aqueous outflow [42]. Together, these mechanisms illustrate how the impaired coagulation regulation in PC deficiency initiates a cascade of ischemic, inflammatory, and proliferative events that can ultimately lead to irreversible vision loss.

Although ocular complications in patients with PC deficiency have traditionally been interpreted as consequences of thrombosis, accumulating evidence now supports a broader role for the PC/APC system in retinal homeostasis. Clinical and experimental studies demonstrate that APC is actively transported from the systemic circulation into the retina and that retinal APC levels are dynamically regulated in response to pathological stress, including proliferative diabetic retinopathy [11,43]. Further studies are required to define the physiological significance of this pathway, its regulation across disease stages, and its contribution to retinal health and pathology.

### 4.1. Neonatal Presentations

In infants with congenital PC deficiency, ocular findings may be severe and can be the first indication of the disorder. Leukocoria (white pupillary reflex) is frequently the presenting symptom that brings affected infants to medical attention. This occurs due to extensive vitreous hemorrhage and tractional retinal detachment that develops in utero or within the first days of life [7,36,39]. Retinal detachment is a particularly devastating complication, with many infants presenting with total retinal detachment that is deemed inoperable. The retinal pathology is characterized by extensive fibrovascular proliferation, vitreous hemorrhage, and tractional membranes that can rapidly progress to complete retinal detachment. Posterior segment pathologies in severe PC deficiency also include persistent fetal vasculature, accompanied by microphthalmia, vitreoretinal dysplasia, and bilateral renal vein thrombosis [44].

Anterior segment abnormalities in congenital PC deficiency include corneal opacities, shallow anterior chambers, lens adhesions, and anterior segment dysgenesis, which may lead to secondary glaucoma [42]. Poor mydriasis and rubeosis iridis may also be present, indicating significant ischemic damage [7,39].

### 4.2. Retinal Vascular Occlusions

In both pediatric and adult patients, PC deficiency is strongly associated with CRVO. Studies have demonstrated that up to 29% of young adults with CRVO have associated activated PC resistance or PC deficiency, compared to 2–7% in the general population. The typical presentation includes venous engorgement, flame-shaped hemorrhages, cotton-wool spots, and optic disc swelling [45,46]. Branch retinal vein occlusion has also been reported, though with somewhat lower frequency than CRVO. The pathogenesis involves thrombosis of retinal venules due to the hypercoagulable state, leading to retinal hypoxia and secondary complications [45,47].

More rarely, combined central retinal artery and vein occlusion can occur, representing an extremely severe manifestation that typically results in devastating visual outcomes. This combination is particularly associated with severe PC deficiency and may be accompanied by carotid artery disease [48,49].

### 4.3. Ischemic Optic Neuropathy

Non-arteritic anterior ischemic optic neuropathy (NAION) has been reported in association with PC deficiency and APC resistance. Some studies have found APC resistance in up to 24% of patients with NAION [50,51], significantly higher than the 5.9% prevalence in control populations, while other studies failed to verify this association [52]. A Factor V Leiden mutation, which confers APC resistance, appears to be a significant risk factor for NAION development [53].

Combined PC and protein S deficiency has been associated with ischemic optic neuropathy, expanding the spectrum of ophthalmic manifestations. The pathogenesis involves thrombosis or hypoperfusion of the short posterior ciliary arteries that supply the optic nerve head [51].

### 4.4. Vitreous Hemorrhage

Vitreous hemorrhage is one of the major ocular complications in severe congenital PC deficiency. This often occurs in conjunction with retinal detachment and may be the result of retinal neovascularization secondary to ischemia or direct hemorrhage from thrombosed retinal vessels. The hemorrhage can be extensive enough to completely obscure fundus details and contribute to leukocoria presentation [7,36,39,54,55].

## 5. The SPCD Mouse: A Breakthrough Model for Studying Retinal APC Transport

Studying the physiological functions of PC has long been hindered by the absence of viable animal models completely lacking the protein. Multiple attempts have been made to generate such models, primarily in mice but also in zebrafish, and a comprehensive summary of these animal models is presented in Table 1. Homozygous PC deficiency (PC^−/^^−^) is embryonically lethal in mice, as all neonates die within 24 h of birth due to severe perinatal consumptive coagulopathy [56]. Subsequent efforts generated transgenic lines expressing residual plasma PC levels ranging from 3% to 18% of normal [57,58]. Although these mice exhibited some prothrombotic and inflammatory manifestations, the residual PC expression limited their suitability for mechanistic transport studies. To overcome the embryonic lethality of complete PC deficiency, our group developed a unique SPCD murine model (PROC^−/^^−^/F8^−^) by combining total PC deficiency with total Factor VIII (F8) deficiency, which causes severe hemophilia A (HA) [59]. This genetic strategy balances the prothrombotic phenotype of PC deficiency with the bleeding tendency of HA, rescuing perinatal lethality and enabling survival into adulthood. Importantly, HA mice (F8^−^) alone display a normal ocular APC phenotype, confirming that the absence of APC in SPCD animals reflects true PC deficiency rather than background genetic effects [11].

One of the SPCD model’s key contributions is demonstrating that retinal PC/APC originates from the systemic circulation rather than from local synthesis. The retina lacks detectable PC mRNA expression yet contains functionally active APC protein under normal conditions [11]. Using SPCD mice completely devoid of endogenous PC, we showed that no PC or APC is detected in the retina at baseline, allowing unambiguous tracking of exogenously administered protein. Following intravenous injection of APC, distinct immunostaining appeared across multiple retinal layers, demonstrating that circulating APC traverses the BRB and accumulates within retinal tissue [11]. These findings establish a direct causal link between systemic APC and its retinal localization, confirming that retinal PC/APC derives from circulating APC/PC which is primarily derived from liver-synthesized protein rather than local production.

## 6. EPCR-Mediated Transport of APC Across the BRB

Given the large molecular size of APC (~62 kDa), its presence within the retina suggests that entry across blood barriers occurs via an active, receptor-mediated process rather than passive diffusion. EPCR resides predominantly within caveolin-1–positive membrane microdomains of endothelial cells [17,62,63]. Ligand binding triggers internalization through caveolin-1–positive vesicles, followed by receptor recycling to the plasma membrane [17,62]. This trafficking mechanism enables transendothelial movement of EPCR-bound ligands and regulates their bioavailability, supporting the concept that EPCR functions as an active transporter rather than a static binding receptor [17].

Using a vascular perfusion technique in mice, Deane et al. demonstrated that the transport of APC across the blood-brain barrier (BBB) is approximately 100-fold higher than that of the extracellular-space marker carbon-14-labeled inulin, indicating a predominantly unidirectional movement from the circulation into the brain [64]. This finding supports the concept of active, receptor-mediated transcytosis for large proteins across the BBB. Blocking the APC-binding site on EPCR inhibited brain entry of APC by more than 85%, and APC uptake was reduced by 64% in EPCR-deficient mice, confirming this receptor’s essential role. Importantly, this translocation of APC was independent of PAR1 activation, and APC analogs with reduced anticoagulant activity (3K3A-APC and 5A-APC) competed for the same EPCR-dependent pathway with comparable affinities.

**Figure 2:**
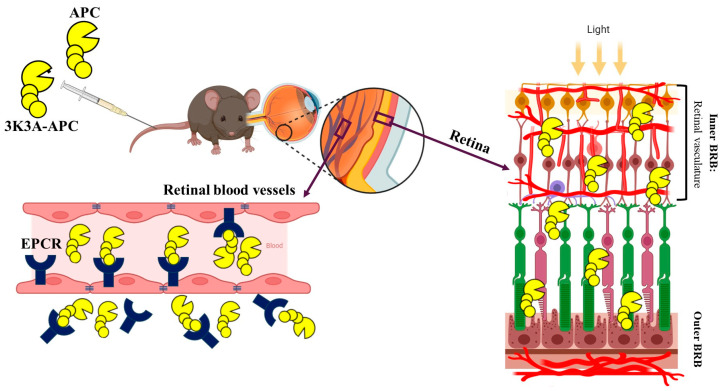
EPCR-mediated transport of APC and 3K3A-APC across the blood–retina barrier following systemic intravenous administration. Schematic illustration of the receptor-mediated pathway by which systemically administered activated protein C (APC) and 3K3A-APC reach the retina. Following intravenous injection, APC circulates through the systemic vasculature and reaches the retinal microcirculation, where it binds the endothelial protein C receptor (EPCR) and undergoes receptor-mediated internalization and transcytosis across the blood–retina barrier (BRB). Within the retina, APC is detected both in co-localization with EPCR and in a free form [11], suggesting availability for direct interaction with retinal cells. Together, these findings support active, EPCR-dependent transport and the feasibility of systemic delivery of APC-based therapeutics to the retina.

Although EPCR has traditionally been considered an endothelial-specific receptor, accumulating evidence demonstrates its expression in a variety of non-endothelial cell types, including neurons, monocytes, Schwann cells, smooth muscle cells, and epithelial cells in various tissues [17,65,66]. This broader expression pattern provides a mechanistic basis for APC’s direct effects on non-endothelial cells, including neuroprotection and modulation of inflammatory cells. In the healthy retina, EPCR is expressed in endothelial cells of the retinal vasculature and is absent from other retinal cell types, including the RPE, photoreceptors, and ganglion cells; its expression is markedly upregulated in models of retinal neovascularization [43]. Song et al. [43] demonstrated that EPCR is essential for normal vascular expansion and branching during retinal development and serves as a primary mediator of pathological neovascularization and vascular leakage under chronic conditions such as proliferative diabetic retinopathy (PDR). Arta et al. provided functional evidence that in the retina, EPCR can mediate active receptor-dependent transport [67]. Using human retinal endothelial cell cultures, they showed that liposomes conjugated with anti-EPCR antibodies exhibited more than a three-fold increase in uptake compared with non-targeted controls. Additionally, encapsulation of corticosteroids within these EPCR-targeted liposomes markedly enhanced anti-inflammatory efficacy. Consistent with these findings, our in vivo studies demonstrated that EPCR functions as a dynamic transporter mediating the translocation of APC and 3K3A-APC from the circulation into the retina (Figure 2) [11]. Importantly, EPCR blockade with neutralizing anti-EPCR antibodies significantly reduced APC accumulation in retinal tissue, confirming that EPCR is required for APC transport into the retina and indicating receptor-mediated transcytosis rather than passive diffusion.

## 7. Therapeutic Potential of APC and 3K3A-APC in Retinal Disease

### 7.1. Preclinical and Clinical Evaluation of APC and 3K3A-APC Signaling

Following the recognition of its cytoprotective and anti-inflammatory properties, APC rapidly evolved from a physiological anticoagulant into a therapeutic candidate for inflammatory and neurovascular diseases. Preclinical studies demonstrated that APC exerts direct protective effects on endothelial and neuronal cells through multiple mechanisms, including anti-apoptotic signaling, endothelial barrier stabilization, and suppression of inflammatory cascades in models of ischemia, stroke, and neurodegeneration [8,9]. These findings established a compelling rationale for translating APC-based therapeutics into clinical evaluation for vascular and neurovascular diseases, including retinal disorders characterized by barrier dysfunction, inflammation, and neurodegeneration.

The development of APC as a therapeutic agent highlighted its primary limitation: potent anticoagulant activity, which poses a substantial bleeding risk. To address this, signaling-selective APC analogs were engineered to preserve the molecule’s cytoprotective and anti-inflammatory functions while markedly reducing its anticoagulant potential. The most extensively characterized analog, 3K3A-APC, incorporates three Lys to Ala substitutions that diminish its ability to inactivate factor Va without disrupting its high-affinity binding to EPCR or its ability to activate PAR1 and PAR3 signaling [8,23]. This selective reduction in anticoagulant function allows safe therapeutic plasma concentrations that are much higher than physiological levels (1–3 ng/mL), typically reaching 45–100 ng/mL or more in clinical trials, without causing significant prolongation of coagulation parameters or increased bleeding risk [9,22,23]. While phase 2 trials of 3K3A-APC in ischemic stroke demonstrated favorable safety [25], recent reports have raised concerns regarding data integrity in some stroke neuroprotection studies [68]. These findings await confirmation in adequately powered phase 3 trials, a consideration relevant to the future development of 3K3A-APC for retinal indications.

Given APC’s proven cytoprotective activity in the central nervous system and its shared vascular and barrier features with the retina, these findings prompted the investigation of its potential role in retinal protection. In ischemic retinal injury models, APC promoted the survival of retinal ganglion cells, prevented apoptosis, and protected axonal integrity [69]. Beyond ischemic injury, APC’s multi-target, pleiotropic actions suggest that it may also mitigate a broad spectrum of retinal disorders driven by chronic inflammation, BRB breakdown, and secondary neurodegeneration.

Neovascular AMD, characterized by choroidal neovascularization (CNV), remains the leading cause of blindness in developed countries, and despite major advances with anti-VEGF therapies, significant limitations persist, including treatment burden, variable response, and progression to fibrosis [70]. These unmet needs provided the rationale for testing APC and its analog 3K3A-APC in preclinical CNV models, where their pleiotropic mechanisms offered potential advantages beyond VEGF inhibition. In laser-induced CNV murine models, APC significantly inhibited the formation of new leakage sites and reversed leakage in 85% of pre-existing lesions, and reduced both lesion depth and total neovascular volume, with efficacy comparable to bevacizumab [71]. Similarly, 3K3A-APC induced regression of established CNV by reducing volume and penetration depth, and it increased the odds of forming non-leaking lesions, underscoring its potent anti-CNV effect [72,73]. Both APC and 3K3A-APC decreased VEGF levels at CNV lesion sites, with the effect of a single intravitreal injection sustained for up to 14 days. A strong correlation was observed between leakage and elevated VEGF, as non-leaking lesions had markedly lower VEGF volumes [72,73]. In parallel, APC stabilized the RPE barrier by inducing translocation of ZO1, a key tight junction protein, to the cell membrane in culture, and similar barrier-stabilizing effects were observed in vivo. The Tie2 receptor partially mediated these actions, as blocking Tie2 abolished APC’s ability to reduce the depth of vascular invasion [71]. Importantly, Minhas et al. demonstrated that APC acts as a direct Tie2 agonist, mimicking angiopoietin-1 binding independently of EPCR or PAR1, thereby stabilizing the vasculature [74].

Beyond its vascular effects, 3K3A-APC reduced inflammatory responses in CNV by significantly decreasing the accumulation and activation of myeloid cells and microglia, and by downregulating NLRP3 inflammasome activity and IL-1β release at the lesion and surrounding retina, thereby attenuating the inflammatory drive that contributes to CNV progression [73]. Collectively, these findings highlight that APC and 3K3A-APC not only suppress CNV but also stabilize retinal barriers and mitigate inflammation, offering a therapeutic profile that addresses multiple pathological mechanisms of neovascular AMD.

In addition, 3K3A-APC has been shown to exert vigorous anti-inflammatory activity in a murine model of lipopolysaccharide (LPS)-induced uveitis, which recapitulates acute inflammatory injury in the eye [75]. Treatment with 3K3A-APC significantly reduced the number of infiltrating leukocytes and inhibited their extravasation from retinal vessels into the parenchyma, thereby limiting tissue damage. Retinal microglia, which normally underwent a marked pro-inflammatory transition after LPS exposure, remained largely quiescent in treated eyes. Notably, the inflammation-associated increase in retinal thickness observed after LPS challenge was substantially diminished by 3K3A-APC, indicating structural preservation of the retina. At the molecular level, the therapy suppressed inflammasome activation, as evidenced by reduced NLRP3 expression and decreased IL-1β levels [75]. These findings demonstrate that 3K3A-APC not only attenuates leukocyte recruitment and microglial activation but also interrupts inflammasome-driven inflammatory cascades, highlighting its potential as a therapeutic approach for retinal diseases with an inflammatory component.

The transition of APC’s retinal protective actions from bench to bedside has commenced. In Japan, a pilot clinical study in patients with ischemic CRVO demonstrated that intravitreal APC treatment is safe and promotes reperfusion of ischemic areas, reduces macular edema, and improves visual acuity [76]. Long-term follow-up confirmed sustained improvements in retinal perfusion and vision, with markedly fewer injections compared to anti-VEGF therapy and no reported safety concerns [10].

Although the pilot and extension studies provide encouraging translational signals, these findings should be interpreted with caution. The cohort was small, conducted at a single center, lacked a randomized control arm, and allowed additional rescue therapies during follow-up, factors known to limit causal inference and precise estimation of treatment effects in early-phase clinical investigations [77,78,79]. Validation in adequately powered, controlled trials is therefore warranted.

Table 2 synthesizes mechanistic information derived from retinal experimental models. It specifies which biological effects were demonstrated with APC and which were evaluated using 3K3A-APC. Direct retinal validation is indicated where available, whereas pathways supported primarily by CNS data are clearly identified as extrapolative.

Notably, APC signaling in the retina is unlikely to follow a strictly sequential model. Rather, EPCR-mediated transcytosis, endothelial PAR1/PAR3 engagement, β-arrestin–biased cytoprotective signaling, and modulation of vascular stabilization pathways may occur in parallel and in a context-dependent manner. APC bound to EPCR at the endothelial surface can initiate PAR-dependent signaling locally, whereas transcytosed APC within the retinal compartment may directly or indirectly influence neuronal and glial responses through secondary mediators. These processes likely operate as an integrated signaling network, with overlapping and temporally dynamic contributions that remain to be fully delineated in retinal tissue.

**Figure 3:**
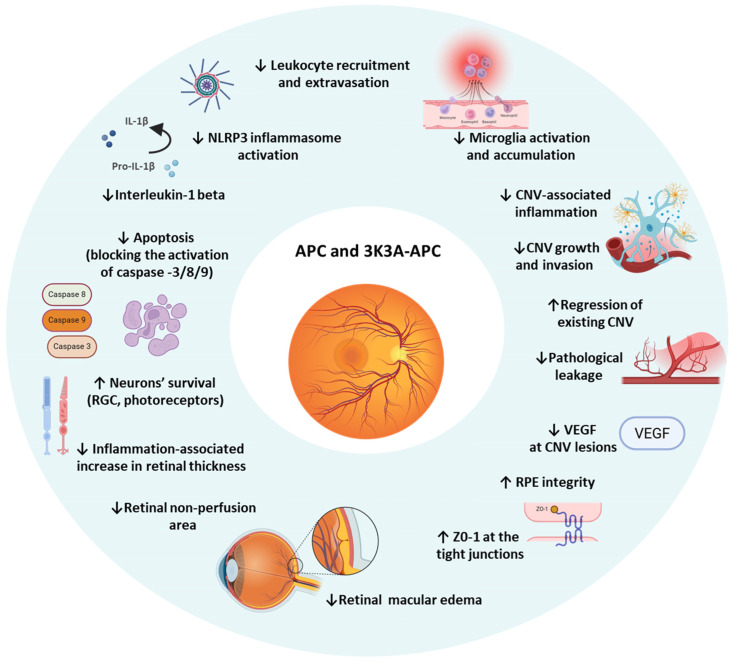
Pleiotropic cytoprotective effects of APC and 3K3A-APC in the retina. Activated Protein C (APC) and its signaling-selective analog 3K3A-APC exhibit broad cytoprotective activity in retinal tissue, including anti-inflammatory, endothelial barrier-stabilizing, anti-angiogenic, and neuroprotective effects. Experimental studies across various retinal models demonstrated that these APCs inhibit microglial activation, leukocyte extravasation, NLRP3 inflammasome activity, and IL-1β release, thereby reducing retinal edema and thickness. At the vascular level, These APCs decrease VEGF levels and CNV growth, promote regression of pre-existing lesions, and stabilize the blood–retina barrier (BRB) by enhancing RPE integrity and increasing ZO-1 translocation to tight junctions. Under ischemic conditions, these APCs suppress apoptosis by inhibiting caspase-3, -8, and -9 activation, thereby preserving neuronal survival in retinal ganglion cells (RGCs) and photoreceptors, limiting retinal non-perfusion areas, reducing macular edema (ME) and improving retinal function. Collectively, APC and 3K3A-APC provide broad cytoprotective activity targeting angiogenic, inflammatory, and neurodegenerative mechanisms in retinal disease.

A schematic summary of the pleiotropic, multi-target cytoprotective, anti-inflammatory, barrier-stabilizing, anti-angiogenic, and neuroprotective mechanisms for APC’s cell signaling effects is presented in Figure 3.

Abbreviations: APC, Activated Protein C; 3K3A-APC, engineered Activated Protein C analog with three Lys → Ala substitutions; BRB, blood–retina barrier; RPE, retinal pigment epithelium; ZO-1, Zonula Occludens-1; VEGF, vascular endothelial growth factor; CNV, choroidal neovascularization; NLRP3, NACHT LRR and PYD domain-containing protein 3; IL-1β, interleukin-1 beta; RGC, retinal ganglion cell; RNP, retinal non-perfusion; ME, macular edema.

### 7.2. Intraocular vs. Systemic Administration of APC and 3K3A-APC for Retinal Disease

The retina is uniquely vulnerable to hemorrhagic complications, as even limited intraocular or intra-retinal bleeding can cause significant and potentially irreversible visual consequences. This vulnerability confers a clear therapeutic advantage to 3K3A-APC over APC for ocular applications. Importantly, preclinical and clinical studies in the CNS and other tissues have demonstrated that both molecules share similar pharmacokinetic profiles and 3K3A-APC retains normal cell-signaling activities, including equivalent EPCR binding, PAR1/PAR3 activation, and cytoprotective effects, while exhibiting >90% reduced anticoagulant activity [8,9,23,24]. Thus, 3K3A-APC preserves full cytoprotective activity while eliminating bleeding risk, a critical advantage for retinal indications.

From a translational perspective, the route of administration is a critical determinant of the clinical feasibility of APC-based therapies. Intraocular delivery, while ensuring high local drug concentrations with minimal systemic exposure, remains an invasive approach that requires repeated injections and is associated with cumulative procedural risks, including patient burden, retinal detachment, and endophthalmitis. These limitations are particularly relevant for chronic retinal diseases with inflammatory or neovascular components that require long-term treatment. At the ocular level, a pilot clinical study demonstrated that intravitreal APC injection is safe and efficacious in patients with ischemic CRVO, promoting reperfusion, reducing macular edema, and improving visual acuity with sustained benefits and markedly fewer injections than anti-VEGF therapy [10]. This provides proof-of-concept for local APC delivery in human retinal disease.

In contrast, systemic administration represents a highly attractive alternative, as it is non-invasive, enables treatment of bilateral retinal disease, and has the potential to confer broad vascular and neuroprotective benefits beyond the eye. Our recent findings demonstrate that systemically administered APC and 3K3A-APC can cross the BRB via EPCR-mediated transport, accumulate in retinal tissue, and induce cytoprotective activities [11], providing proof of concept support for the systemic delivery approach in retinal disease.

While most current treatments for posterior segment pathology rely on intravitreal injections, systemic drug delivery for retinal diseases is an established therapeutic approach. Several systemic medications have demonstrated efficacy in modifying diabetic retinopathy progression, and there is growing interest in developing oral therapies for retinal diseases to reduce the burden of repeated intravitreal injections, with multiple agents currently under investigation for AMD, diabetic retinopathy, and other conditions [80,81,82,83].

For APC-based therapies, systemic delivery presents specific pharmacological challenges. Both APC and 3K3A-APC have short plasma half-lives of approximately 12–20 min in humans, reflecting rapid inactivation through complex formation with serine protease inhibitors, primarily protein C inhibitor (PCI), α1-antitrypsin, and α2-macroglobulin, followed by clearance from circulation [24,84,85]. This necessitates consideration of dosing frequency for sustained therapeutic effect. In clinical trials for ischemic stroke, 3K3A-APC was administered intravenously at doses up to 540 μg/kg, with a favorable safety profile and no evidence of accumulation [24,25]. The short systemic half-life may be partially offset by EPCR-mediated transcytosis across the BRB, which could concentrate APC and 3K3A-APC within the retinal compartment [11]. Furthermore, the prolonged functional activity of APC in the retina, with anticoagulant activity detected 24 h post-injection [11] compared to a plasma half-life of only 12–20 min, suggests that major plasma APC inhibitors may be absent or less active in the retinal microenvironment. However, the specific pathways governing APC degradation or clearance within the retina have not been directly investigated and remain an important area for future research. Thus, the optimal dosing regimen for retinal indications, whether systemic or intravitreal, remains to be determined in future clinical trials.

Notably, recent advances in dermatology, where topical 3K3A-APC is currently under clinical evaluation for chronic wound healing [86], further underscores the therapeutic versatility of localized APC delivery. Taken together, these observations highlight that both systemic and local routes hold promise for APC-based retinal therapy. The optimal strategy will depend on balancing efficacy, safety, accessibility, expense, and patient burden, and further studies are needed to pave the way for tailored interventions that harness APC’s pleiotropic protective actions for retinal disease.

## 8. Limitations and Conclusions

This review has several limitations. Much of the mechanistic understanding of APC signaling in the retina is extrapolated from studies in other vascular beds and the central nervous system, as direct retinal evidence for specific receptor interactions remains limited. Preclinical efficacy data are derived predominantly from murine models, which may not fully recapitulate human retinal disease complexity. The optimal dosing regimen, route of administration, and long-term safety profile of APC and 3K3A-APC for retinal indications remain to be established in future clinical trials.

Despite these limitations, this review delineates the multifaceted role of the PC pathway in the retina, encompassing both its physiological functions and its therapeutic potential at pharmacological concentrations.

The development of a murine model with complete PC deficiency has enabled, for the first time, exploration of the physiological role of PC in the retina [11]. Together with recently published data highlighting the role of the APC–EPCR axis in retinal pathologies [43], accumulating evidence underscores the pivotal contribution of PC and APC to retinal homeostasis and reveals the context-dependent duality of EPCR in retinal biology. These insights position the APC–EPCR axis not only as a guardian of retinal integrity but also as a novel therapeutic gateway for treating multifactorial retinal diseases.

A key mechanistic discovery is the physiological recognition that EPCR mediates the transport of APC across the BRB. This receptor-mediated transcytosis mechanism enables exogenously administered therapeutic APC and 3K3A-APC to enter the retina, remain biologically active, and confer potent cytoprotective effects on retinal tissue [11]. This discovery identifies EPCR as a receptor-mediated pathway that facilitates the translocation of APC across neural–vascular barriers and provides mechanistic support for the feasibility of systemic delivery of APC-based therapeutics to the retina.

From a translational standpoint, the fact that 3K3A-APC has been engineered to eliminate bleeding risk while preserving cytoprotective activity allows its safe use at therapeutic concentrations, making both local and systemic routes of APC-based therapy viable [8,23].

This review integrates our findings with the broader literature on retinal vascular biology and emerging therapeutic approaches for retinal diseases. Several therapeutic molecules currently in development target pathways intersecting with APC signaling, including faricimab (VEGF-A/Ang-2) [87,88], Tie2 activators [89], and PAR1 modulators [90], suggesting potential for complementary therapeutic approaches. Future research should define the conditions under which the physiological functions of the PC/APC system can be therapeutically leveraged, optimizing concentration, timing, and disease context to maximize efficacy and safety while expanding clinical accessibility. In summary, this review summarizes extensive insights into the therapeutic advantages of the APC pathway in retinal disease and supports the continued development of APC and 3K3A-APC as pleiotropic, multi-target therapeutics for blinding retinal disorders.

## Figures and Tables

**Table 1:** Summary of Animal Models for PC Deficiency.

Animal	Model/Genotype	PC Level (% of WT)	Fate/Survival	Phenotype	Method of Mutation/Deficiency	Technique of Model Generation
Mice [56]	PC^−/^^−^ (Total Deficiency)	0%	Embryos develop normally until E17.5; all neonates died within 24 h after birth.	Lethal perinatal consumptive coagulopathy with microvascular thrombosis in the brain and hepatic necrosis. Severe bleeding near fibrin deposition; no plasma fibrinogen detected.	Targeted inactivation of the murine Proc locus replacing the entire coding region with a neo^R^ cassette.	Homologous recombination in embryonic stem (ES) cells replacing the entire murine Proc coding region with a neomycin-resistance cassette.
Mice [57,58]	PC^−/^^−^ and PC^−/^^−^ (PCTg535–PCTg527)	1–18%	PC^−/^^−^: perinatal lethal; transgenic PCTg lines survive to adulthood depending on PC level.	Severe perinatal coagulopathy in nulls; PCTg lines show spontaneous thrombosis, necrosis (paws, ear, face), and inflammation (increased IL-6, WBCs).	Targeted deletion of PC gene with transgenic rescue using mouse PC cDNA under the Factor X promoter on a PC^−/^^−^ background.	Targeted deletion via homologous recombination combined with transgenic insertion using a cosmid-based construct of murine PC cDNA under the FX promoter.
Mice [59]	PC^−/^^−^/F8^−^	0% (severe deficiency)	Survive to adulthood; Factor VIII deficiency rescues perinatal lethality of PC deficiency.	Double mutant exhibits balanced hemostasis, enabling in vivo investigation of PC/APC; normal development, viable for long-term studies.	Targeted double knockout of PROC and F8 genes.	Double targeted knockout generated by CRISPR/Cas9 and homologous recombination, deleting Proc and F8 loci.
Mice [60]	PROC c.1198G>A (p.Gly400Ser) (heterozygous)	Reduced Activity normal antigen levels	Survive to adulthood; homozygous mice not obtained.	Type II pattern deficiency with secondary consumptive coagulopathy; liver steatosis, splenic congestion, fibrin deposition, and inflammation.	CRISPR/Cas9 point mutation introducing the PROC c.1198G>A (p.Gly400Ser) variant.	CRISPR/Cas9 genome editing introducing a single-nucleotide knock-in mutation c.1198G>A (p.Gly400Ser) in the PROC locus.
Zebrafish [61]	PC^−/^^−^ (Total deficiency, dual-locus deletion)	≈0%	Survive to adulthood; ~70% lethality by 1 year.	Spontaneous thrombotic coagulopathy (92.5% larvae); inflammation with il1b upregulation and neutrophil migration defects.	CRISPR/Cas9 genome editing generates a 17.3-kb deletion, ablating both proc loci.	CRISPR/Cas9 genome editing generates a 17.3-kb deletion, removing both duplicated proc loci.

**Table 2:** Mechanistic Integration of Retinal Effects of Pharmacological APC and 3K3A-APC.

Retinal Effect/Action	Likely Pathway/Mediator	Molecule Tested	Evidence Level (Retina)
Systemic BRB Entry	EPCR-mediated transcytosis	APC; 3K3A-APC	Demonstrated (in vivo murine); [11]
CNV Regression	Multifactorial: Tie2 stabilization; VEGF reduction; inflammatory suppression	APC; 3K3A-APC	Demonstrated (in vivo murine); [71,72,73]
Reduction of VEGF Expression in CNV	Tie2-associated vascular stabilization; inflammatory modulation	APC; 3K3A-APC	Demonstrated (in vivo murine); [72,73]
RPE Barrier Stabilization	Tie2 signaling; ZO-1 redistribution	APC	Demonstrated (in vitro; supportive in vivo); [71,74]
Inflammasome Suppression	Reduced NLRP3/IL-1β	3K3A-APC	Demonstrated (in vivo murine); [73,75]
Microglia Modulation	Reduced activation phenotype	3K3A-APC	Demonstrated (in vivo murine); [73,75]
Neuroprotection (Ischemic Injury)	Reduced caspase-3/-8/-9 activation	APC	Demonstrated (in vivo murine); [69]
PAR Signaling Hierarchy	PAR1/PAR3 interaction	CNS-based evidence	Not directly tested in retina; [8,9,19,23]
β-arrestin 2–dependent signaling	Biased PAR1 signaling	CNS-based evidence	Not directly tested in retina; [18,19,20,21,22]

Abbreviations: BRB, blood–retina barrier; EPCR, endothelial protein C receptor; CNV, choroidal neovascularization; VEGF, vascular endothelial growth factor; RPE, retinal pigment epithelium; NLRP3, NOD-like receptor family pyrin domain containing 3; PAR, protease-activated receptor.

## Data Availability

No new data were created or analyzed in this study. Data sharing is not applicable to this article.
